# Pollinator size and its consequences: Robust estimates of body size in pollinating insects

**DOI:** 10.1002/ece3.4835

**Published:** 2019-02-07

**Authors:** Liam K. Kendall, Romina Rader, Vesna Gagic, Daniel P. Cariveau, Matthias Albrecht, Katherine C. R. Baldock, Breno M. Freitas, Mark Hall, Andrea Holzschuh, Francisco P. Molina, Joanne M. Morten, Janaely S. Pereira, Zachary M. Portman, Stuart P. M. Roberts, Juanita Rodriguez, Laura Russo, Louis Sutter, Nicolas J. Vereecken, Ignasi Bartomeus

**Affiliations:** ^1^ School of Environmental and Rural Science University of New England Armidale New South Wales Australia; ^2^ CSIRO Agriculture Brisbane Queensland Australia; ^3^ Department of Entomology University of Minnesota St. Paul Minnesota; ^4^ Agroscope, Agroecology and Environment Zürich Switzerland; ^5^ School of Biological Sciences & Cabot Institute University of Bristol Bristol UK; ^6^ Departamento de Zootecnia—CCA Universidade Federal do Ceará Fortaleza Brazil; ^7^ Animal Ecology and Tropical Biology, Biocenter University of Würzburg Würzburg Germany; ^8^ Dpto. Ecología Integrativa Estación Biológica de Doñana (EBD‐CSIC) Sevilla Spain; ^9^ School of Agriculture, Policy and Development The University of Reading Reading UK; ^10^ Australian National Insect Collection, CSIRO Canberra Australian Capital Territory Australia; ^11^ Botany Department Trinity College Dublin Dublin Ireland; ^12^ Interfaculty School of Bioengineers, Université Libre de Bruxelles Bruxelles Belgium

**Keywords:** Apoidea, biogeography, body size, dry weight, pollimetry, pollination, predictive models, *R* package, Syrphidae

## Abstract

Body size is an integral functional trait that underlies pollination‐related ecological processes, yet it is often impractical to measure directly. Allometric scaling laws have been used to overcome this problem. However, most existing models rely upon small sample sizes, geographically restricted sampling and have limited applicability for non‐bee taxa. Allometric models that consider biogeography, phylogenetic relatedness, and intraspecific variation are urgently required to ensure greater accuracy. We measured body size as dry weight and intertegular distance (ITD) of 391 bee species (4,035 specimens) and 103 hoverfly species (399 specimens) across four biogeographic regions: Australia, Europe, North America, and South America. We updated existing models within a Bayesian mixed‐model framework to test the power of ITD to predict interspecific variation in pollinator dry weight in interaction with different co‐variates: phylogeny or taxonomy, sexual dimorphism, and biogeographic region. In addition, we used ordinary least squares regression to assess intraspecific dry weight ~ ITD relationships for ten bees and five hoverfly species. Including co‐variates led to more robust interspecific body size predictions for both bees and hoverflies relative to models with the ITD alone. In contrast, at the intraspecific level, our results demonstrate that the ITD is an inconsistent predictor of body size for bees and hoverflies. The use of allometric scaling laws to estimate body size is more suitable for interspecific comparative analyses than assessing intraspecific variation. Collectively, these models form the basis of the dynamic *R* package, “*pollimetry,*” which provides a comprehensive resource for allometric pollination research worldwide.

## 1 INTRODUCTION

Body size is an important functional trait that influences ecological patterns across all levels of biological organization. In insects, adult body size variation is the outcome of natural selection affecting physiological and biochemical processes during ontogeny (Chown & Gaston, 2010). Body size impacts metabolic and growth rates (Angilletta, Steury, & Sears, 2004; Ehnes, Rall, & Brose, 2011), life history (e.g., lifespan and reproductive rate; Speakman, 2005, Teder, Tammaru, & Esperk, 2008) and ecological attributes, such as species abundance, trophic interactions, geographic range size, and dispersal ability (Brown, Gillooly, Allen, Savage, & West, 2004; DeLong et al., 2015; Stevens, Trochet, Dyck, Clobert, & Baguette, 2012; Velghe & Gregory‐Eaves, 2013; White, Ernest, Kerkhoff, & Enquist, 2007). In addition, body size can drive key ecosystem functions and services such as decomposition, carbon cycling, predation, primary productivity, and pollination (Garibaldi et al., 2015; Greenleaf, Williams, Winfree, & Kremen, 2007; Rudolf & Rasmussen, 2013; Schramski, Dell, Grady, Sibly, & Brown, 2015; Woodward & Hildrew, 2002).

Body size is most commonly measured as specimen dry weight. As such, obtaining direct measurements can be impractical. First, dehydrating and weighing pinned specimens is time‐consuming and involves intensive handling of the specimens, increasing the likelihood of damage. Second, the collection process may affect a specimen's final weight, especially if specimens are damaged internally (e.g., rotten material) or externally (e.g., loss of appendages) (Rogers, Buschbom, & Watson, 1977, Henschel & Seely, 1997, but see Gilbert, 2011). Allometric scaling laws can be used to overcome these problems. These laws refer to how traits, which can be morphological, physiological or chemical, co‐vary with an organism's body size, often with important ecological and evolutionary implications (Gould, 1966). Hence, these scaling laws can be utilized to estimate body size, using an easy to measure morphological trait and therefore circumventing the use of problematic direct measurements of body size.

Equations which utilize allometric scaling to predict body size as a function of a co‐varying morphological trait have emerged across many biological disciplines. The most commonly used co‐varying trait used to predict body size is body length, having been used extensively in fish (Karachle & Stergiou, 2012), mammals (Trites & Pauly, 1998) and both aquatic (Burgherr & Meyer, 1997) and terrestrial invertebrates (Rogers et al., 1977; Sabo, Bastow, & Power, 2002). These models often show considerable predictive power at the ordinal level (*R^2^* > 0.9), which has led to the proliferation of multiple models for a wide range of taxa (e.g., there are 26 body size ~ body length models for Diptera—See Supporting Information Appendix S1). However, when compared, these models show considerably different allometric scaling coefficients both within‐ and between insect orders (Brady & Noske, 2006; Sample, Cooper, Greer, & Whitmore, 1993; Schoener, 1980). Previously, these differences have been attributed to biogeographic factors, such as latitude (Martin, Proulx, & Magnan, 2014) and/or methodological influences such as sampling biases (e.g., the range of sampled body sizes, Sage, 1982). Importantly, they have also notably failed to incorporate sexual size dimorphism which is common in invertebrates (Shreeves & Field, 2008).

The allometry of functional traits has been shown to influence plant–pollinator interactions, specifically in bees. For example, smaller body size is associated with higher activity periods in response to available light (Streinzer, Huber, & Spaethe, 2016), whereas larger body size is related to greater pollen load capacity (e.g., within *Melipona quadrifasciata* colonies, see Ramalho, Imperatriz‐Fonseca, & Giannini, 1998) as well as greater interspecific foraging distances (Greenleaf et al., 2007). Importantly, body size can influence and constrain plant–pollinator interactions and trait matching both within and between pollinator groups (Bartomeus et al., 2016; Stang, Klinkhamer, Waser, Stang, & Meijden, 2009). Therefore, allometric traits central to pollination‐related ecological processes appear and interact at the intra‐ and interspecific levels. Despite their ubiquity, few predictive models for body size exist for pollinating insects below the ordinal level, with one notable exception. Cane (1987) pioneered a predictive model for bee body size as a function of the intertegular distance (ITD) (the distance between the wing‐attachment points on either side of the thorax (see Supporting information Figure S1A). Cane's model identified the ITD as an important body size proxy which has since been used to establish other ecologically important allometric relationships, primarily at the interspecific level (e.g., foraging distances, bee proboscis length and wing loading; Greenleaf et al., 2007; Cariveau et al., 2016a; Bullock, 1999).

The robustness of the ITD as a body size predictor has not been properly tested across a wide range of taxa. First, the original model is based solely on 20 North American solitary bee species. Second, differential allometric coefficients have been observed with other species (Bullock, 1999). Third, the accuracy of intraspecific body size estimation from the ITD has not been assessed extensively, except within *Bombus *spp. (Hagen & Dupont, 2013) and *Osmia* spp. (Bosch & Vicens, 2002; Rust, 1991). Fourth, sexual size dimorphism, present in 80% of Aculeata (Shreeves & Field, 2008), can lead to differential coefficients in determining body size in male and females of the same species (e.g., *Osmia lignaria propinqua*, Bosch & Vicens, 2002), highlighting the need to include sex‐specific co‐variation. Fifth, body size variation has been repeatedly linked to phylogeny, compelling allometric studies to incorporate species’ evolutionary histories (Blomberg, Garland, & Ives, 2003; Garland & Ives, 2000). Lastly, other key pollinating taxa, such as hoverflies (Diptera: Syrphidae) lack allometric models.

These knowledge gaps are largely due to the lack of: (a) a general repository to house and connect all relevant allometric models; (b) large high resolution datasets to build more accurate models that can incorporate co‐variates and (c) an iterative framework, such as those utilized in ecological forecasting (Dietze et al., 2018; Harris, Taylor, & White, 2018) to continuously update existing models with new datasets, methodologies, and technologies. Addressing these key deficiencies will increase model accuracy and the applicability of allometric scaling to pollinating insects.

Here, we catalogue pre‐existing body size ~ trait models for key pollinating insect taxa (Diptera, Hymenoptera and Lepidoptera) and develop new models within an iterative framework for two focal pollinating taxa: bees and hoverflies, which incorporate species evolutionary histories, intraspecific variation and biogeography. These form the basis of a new *R* package, entitled “*pollimetry.*” Specifically, we address the following research questions:

1. Is ITD a robust predictor of interspecific body size variation for two dominant pollinator taxa, bees, and hoverflies?
2. Does incorporating biogeographic region, phylogenetic or taxonomic relatedness and sexual dimorphism improve interspecific predictions of pollinator body size measured as the ITD?
3. Is ITD reliable in predicting intraspecific variation in both bees and hoverflies and what sample size is required to accurately estimate intraspecific body size and ITD values?

## 2 MATERIALS AND METHODS

### 2.1 Pre‐existing models

We collated 26 body size ~ trait models for Diptera, 38 for Hymenoptera and 21 for Lepidoptera groups. We also gathered nine equations for bee foraging distance from two sources (Greenleaf et al., 2007; van Nieuwstadt & Iraheta, 1996), as well as allometric models for estimating bee tongue length (Cariveau et al., 2016a, 2016b), bee wing loading (Bullock, 1999) and total nectar load (Henry & Rodet, 2018; see Supporting Information Appendix S1).

### 2.2 Specimen collection and measurements

We obtained bee and hoverfly specimens from recent field research projects on insect pollinator diversity. We included studies across four continents. In Australia, collections were made in New South Wales, Victoria, Queensland, South Australia, and the Northern Territory. In Europe, we amassed specimens from Belgium, Germany, Ireland, Spain, Switzerland, and the United Kingdom. In the Americas, we included collections from Minnesota, USA and Ceará, Brazil.

The majority of specimens were processed within three to six months of collection, although some, in particular, those from Victoria, Australia, Belgium, Switzerland were of variable ages: ranging from one to five years since collection. We excluded damaged specimens. Except for corbiculate bees, pollen loads were not removed prior to measurement. In addition, Cane (1987)'s) original data from Alabama, USA was obtained using Engauge Digitizer version 10.6 (Mitchell, Muftakhidinov, Winchen, & Jędrzejewski‐Szmek, 2017). For every specimen, we obtained sample location (latitude and longitude) and taxonomic identity. Full information about specimen identification (and taxonomic resources) and deposition locations are provided in the Supporting Information Appendix S1.

In total, we measured 391 bee species (4,035 specimens) from Australia, Europe, North America, and South America and measured 103 hoverfly species (399 specimens) from Australia and Europe (see Supporting Information Appendix S1). Six out of seven bee families (all except Stenotritidae) and three out of four hoverfly subfamilies (all except Microdontinae) were represented. The mean specimen number per bee species was nine (♀) and five (♂) and ranged from 1–201. In hoverflies, the mean specimen number per species was three for both sexes and ranged from 1–50.

### 2.3 Body size, intertegular distance, and body length

Body size was measured as the dry weight in milligrams of each specimen. We therefore refer to body size as dry weight herein for continuity. Specimens were first dehydrated at 70°C for at least 24 hr to remove residual humidity and then weighed on an analytical balance to an accuracy of 0.001 g. All North American bees as well as small‐bodied Australian bees were dehydrated and weighed prior to being mounted on a pin. For all other specimens, pins were not removed prior to weighing. Instead, we identified the pin type and weighed a sample of 10–50 pins per type. The mean weight was then subtracted off the total weight. Pin weight variance was minimal (range of standard errors: 6.3*10^‐4 ^to 2 mg). The Intertegular distance was measured in millimeters using a stereo‐microscope, either mounted with a calibrated scale or microscope camera. Body length (BL) was measured along the lateral side of each specimen with a calibrated scale or microscope camera for Australian, British, German, Irish, and Spanish specimens (see Supporting Information Appendix S1 for visual representation of trait measurements). BL was defined as the total length from the point of antennal insertion to the terminal abdominal tergite (as in Supporting information Figure S1B) or for bent specimens, as the sum of the head, thorax, and abdomen.

### 2.4 Data analysis: model structures

All analyses were undertaken in *R* (version 3.5.1) (R Core Team, 2018). We first assessed the Pearson's correlation coefficient between the ITD and BL using species’ mean values. The ITD and BL were highly correlated in both bees (*r* = 0.95), and hoverflies (*r* = 0.85). We then compared both the ITD and BL independently in predicting dry weight using ordinary least squares (OLS) regression to select the best predictor. For these analyses, we used species’ means. The ITD was marginally more predictive than BL in estimating dry weight in bees (ITD *R^2^*: 0.93; BL *R^2^*: 0.92) and considerably better than BL for hoverflies (ITD *R^2^*: 0.81; BL *R^2^*: 0.72). Most importantly, the ITD is easier to measure unambiguously than BL. Hence, we used the ITD in the following analyses.

As traditionally performed, we used log‐transformed values in the model formulation because allometric relationships are typically described by a power function (*y* = *ax^b^*) which is linearized when log‐transformed:

ln(y)=ln(α)+β×ln(x)

where *y* = dry weight, *α* = intercept, *β* = allometric coefficient and *x* = ITD.

We specified Bayesian generalized linear mixed models (GLMM) with the *brms *package (version 2.5.0) (Bürkner, 2017). Dry weight was predicted as a function of the ITD in interaction with sex and taxonomic grouping: bee families following Michener (2007) and hoverfly subfamilies following Mengual, Ståhls, and Rojo (2015). Bayesian GLMMs allowed us to use all individual specimens’ measurements by including a nested random effect: *species *were nested within their *biogeographic region of origin*. A few specimens from five bee species were removed from their introduced ranges (in parentheses) prior to analyses: *Andrena wilkella *(North America), *Halictus rubicundus* (North America), *Lasioglossum leucozonium *(North America), *Anthidium manicatum *(North America), and *Apis mellifera *(Australia). We call these models taxonomic GLMMs. Both bee and hoverfly models were run for 2000 iterations with a burn‐in of 1,000. We set Δ to 0.99 and manipulated maximum tree depth between 10 and 20 for individual models to avoid divergent transitions. We fitted each model with weakly informative priors based on our domain expertise; priors are explicitly provided in accompanying R code. Chain convergence was assessed using the $\hat{R}$ statistic (See Data Availability) (Gelman & Rubin, 1992). Posterior predictive checks were visualized using the *bayesplot* package (version 1.6.0, Gabry & Mahr, 2017).

### 2.5 Data analysis: incorporating phylogeny

We explored the influence of phylogenetic relatedness in predicting dry weight for bees only because a well‐resolved hoverfly phylogeny was not available. We constructed an applicable phylogeny for our dataset using a bee genera backbone tree (Hedtke, Patiny, & Danforth, 2013). We removed nonrepresented genera using the *ape* package (version 5.1, Paradis, Claude, & Strimmer, 2004). Species tips were added to genera nodes as polytomies of equal branch length relative to the genera branch length using the *phytools* package (version 0.6‐44, Revell, 2012). This excluded a total of three species whose genera weren't included in Hedtke et al. (2013)’s phylogeny: *Flavipanurgus venustus*, *Protomeliturga turnerea, *and *Tetrapedia diversipes*. As the infrageneric polytomies add an artificial element to the phylogeny, we made the explicit assumption that phylogenetic patterns in body size were assessed at and above the genus level.

**Figure 1:**
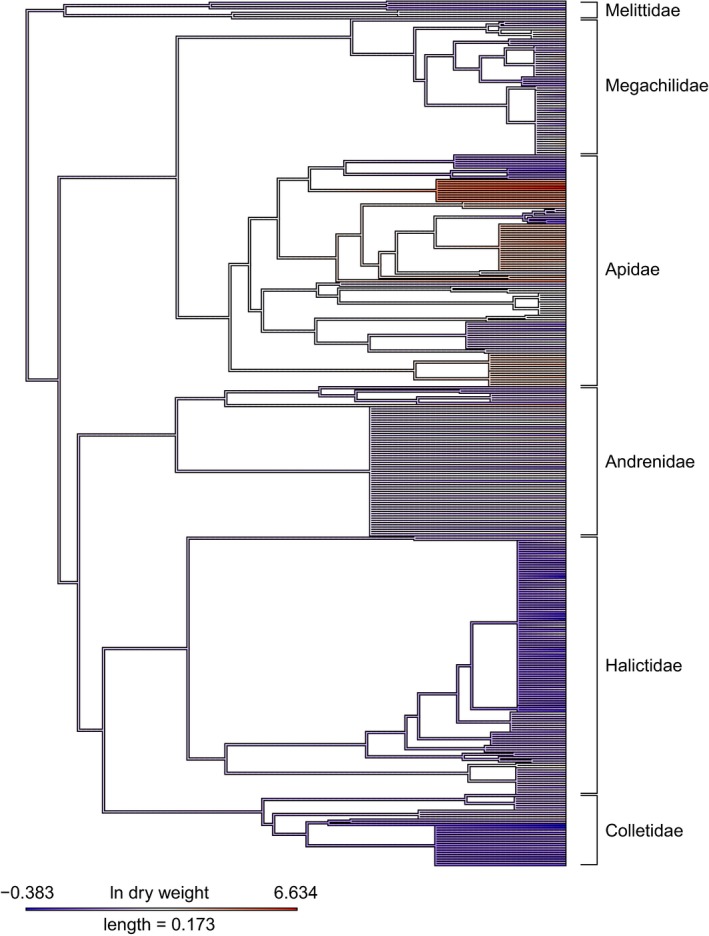
Chronogram of bee genera (data from Hedtke et al., 2013) with infrageneric species polytomies. Branch lengths correspond to relative time since divergence. Color denotes mean ln dry weight (mg) of each bee species

We fitted a chronogram from our phylogeny by penalized likelihood using a correlated rate model with the *ape* package (version 5.1, Paradis et al., 2004). We then assessed the significance of phylogenetic signal using Pagel's λ, using the mean log‐transformed dry weight of each species (Pagel, 1999) with the *phytools* package (version 0.6‐44, Revell, 2012). We found a highly significant signal in bee *dry* weight (λ: 0.846, *p* < 0.001) (Figure 1). Therefore, we implemented a nested phylogenetic generalized linear mixed model (PGLMM), which considered ITD in interaction with intraspecific sexual dimorphism while accounting for phylogenetic dependencies with a nested random term: species nested within region (i.e., the nested species term was constrained by the constructed phylogeny). We refer to these models as phylogenetic GLMMs.

### 2.6 Data analysis: model selection: Bayesian R^2^ and K‐fold cross‐validation

We first fitted the two full models described above: a taxonomic GLMM and a phylogenetic GLMM. To assess their predictive precision, we compared these models with reduced models (i.e., without sex or taxonomy as either intercepts/slopes, see Table 1) including the random term along with two ITD‐only models, with or without the random term in order to select the most suitable models for inclusion in the *R* package. We chose to rank our models based upon their Bayesian *R^2^* and K‐fold cross‐validation (CV) because the Widely applicable information criterion (WAIC) and Leave‐one‐out information criterion (LOO‐IC) were inappropriate due to pWAIC estimates of >0.4 and Pareto *k* estimates of >0.7 (Gelman, Goodrich, Gabry, & Ali, 2017; Vehtari, Gelman, & Gabry, 2017). To undertake *K*‐fold CV, datasets were divided into 10 equal sets containing a random subset of species. Each model was then evaluated iteratively upon each *k*–1 set (training set consisting of nine sets) by comparing the actual and predicted values within the one left out “test” set. This was done repeatedly so each set was both the test set and contained within the training sets from which an information criterion weighting was then calculated.

**Table 1:** Model selection tables for bee and hoverfly interspecific models

Model No.	Taxa	Model type	Model formulae	*R^2^*	K‐CV	Δ	RMSE
**1**	**Bees**	***Taxo. *GLMM**	**ln(Dry weight) ~ ln(ITD) + Family + Sex+ Family:ln(ITD)** **+ Sex:ln(ITD) + (1** **| Region/Species)**	**0.946**	**2763.7**	**0**	**11.313**
**2**			ln(Dry weight) ~ ln(ITD) + Family + Sex + Sex:ln(ITD) + (1 | Region/Species)	0.946	2774.3	10.7	11.216
3			ln(Dry weight) ~ ln(ITD) + Family + Sex + (1 | Region/Species)	0.946	2778.2	14.5	11.629
4			ln(Dry weight) ~ ln(ITD) + Family + Sex + Family:ln(ITD) + (1 | Region/Species)	0.946	2790.9	27.3	11.588
**5**			**ln(Dry weight) ~ ln(ITD) + Sex**	**0.945**	**2803.7**	**30.9**	**11.339**
6			ln(Dry weight) ~ ln(ITD) + Sex + Sex:ln(ITD)	0.945	2834.6	70.9	10.937
**7**			**ln(Dry weight) ~ ln(ITD) + Family + (1** **| Region/Species)**	**0.943**	**2945.3**	**181.7**	**12.092**
8			ln(Dry weight) ~ ln(ITD) + Family + Family:ln(ITD) + (1 | Region/Species)	0.943	2951.5	187.9	12.462
9			**ln(Dry weight) ~ ln(ITD) + (1** **| Region/Species)**	**0.942**	**2985.9**	**222.3**	**11.896**
**10**			ln(Dry weight) ~ ln(ITD)	0.898	4990.2	2226.6	15.565
**1**	**Bees**	***Phylo.* GLMM**	**ln(Dry weight) ~ ln(ITD) + Sex +Sex:ln(ITD) + (1|Region/Species)**	**0.944**	**2882.5**	**0**	**10.228**
**2**			ln(Dry weight) ~ ln(ITD) + Sex + (1|Region/Species)	0.944	2920.3	37.8	10.519
3			ln(Dry weight) ~ ln(ITD) + (1|Region/Species)	0.941	3079.5	197	10.997
**1**	**Hoverflies**	***Taxo.* GLMM**	**ln(Dry weight) ~ ln(ITD) + Sex + Sex:ln(ITD) + (1|Region/Species)**	**0.820**	**520.6**	**0**	**4.747**
**2**			**ln(Dry weight) ~ ln(ITD) + Subf** **+ Sex + (1|Region/Species)**	**0.820**	**531.9**	**11.3**	**4.649**
3			ln(Dry weight) ~ ln(ITD) + Subf + Sex + Sex:ln(ITD) + (1|Region/Species)	0.819	533.3	12.7	4.725
**4**			ln(Dry weight) ~ ln(ITD) + Subf + Sex + Subf:ln(ITD) + (1|Region/Species)	0.820	533.6	13	4.743
**5**			ln(Dry weight) ~ ln(ITD) + Sex + (1|Region/Species)	0.821	537.4	16.8	4.663
6			ln(Dry weight) ~ ln(ITD) + Subf +Sex + Subf:ln(ITD) + Sex:ln(ITD) + (1|Region/Species)	0.819	538.7	18.1	4.896
**7**			**ln(Dry weight) ~ ln(ITD) + (1|Region/Species)**	**0.810**	**544.8**	**24.2**	**4.808**
**8**			**ln(Dry weight) ~ ln(ITD) + Subf** **+ (1|Region/Species)**	**0.810**	**548.2**	**27.6**	**4.801**
**9**			ln(Dry weight) ~ ln(ITD) + Subf + Subf:ln(ITD) + (1|Region/Species)	0.811	552.1	31.5	4.886
10			ln(Dry weight) ~ ln(ITD)	0.762	600.6	80	6.170

Notes

Models in bold are those included in the *R* package. Model types: (a) Taxo. GLMM: taxonomic generalized linear mixed models and (b) Phylo GLMM: phylogenetic generalized linear mixed model. lnITD: ln intertegular distance (mm), Subf: Subfamily, *R^2^*: Bayesian R^2^, K‐CV: K‐fold cross‐validation, Δ: ΔK‐fold CV and RMSE: root‐mean‐square error. Model parameters of the best‐fitting models are shown in Supporting Information Appendix S1.

### 2.7 Model comparisons: Root‐mean‐square error

We assessed the predictive error of all formulated models on the basis of the root‐mean‐square error (RMSE), as it is expressed in the same units of the response variable, between observed‐predicted dry weight values. We also compared these error estimates between our models and predicted values from Cane (1987)’s original model. Lastly, we calculated the RMSE for observed‐predicted values from pre‐existing body length models for both taxa (applicable Diptera and Hymenoptera models for Syrphidae and Apoidea, respectively) using our body length measurements.

### 2.8 Data analysis: intraspecific predictions

We assessed the utility of the ITD in predicting intraspecific dry weight variation. For the 10 most abundant bee species of a given sex (nine using females, one using males) and five most abundant hoverfly species (all using females), we tested the utility of the ITD in predicting intraspecific body size variation using species‐level OLS regression.

To estimate the adequate sample size needed for robust mean trait measures for each bee species, we plotted trait means independently by resampling from one through *n* where *n* = total sample size. We then inferred the adequate sample size whereby variance stabilized within the 95% confidence intervals of the total sample size.

## 3 RESULTS

### 3.1 Interspecific model selection and performance

**Figure 2:**
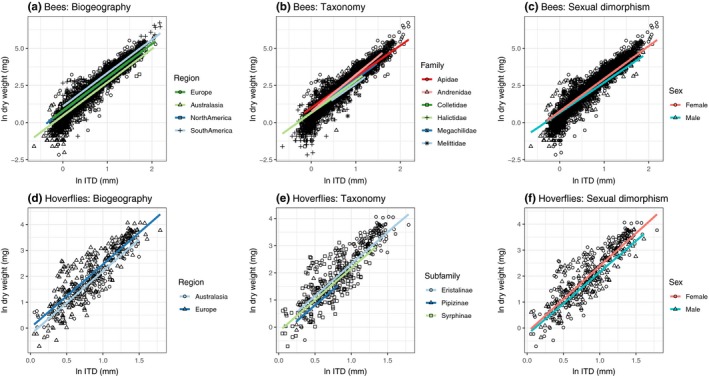
Dry weight (mg) ~ Intertegular distance (ITD) interspecific relationships. From left to right: influence of biogeographic region, taxonomic grouping, and sexual dimorphism. Lines represent the posterior fits from Bayesian generalized linear mixed models. Credible intervals are omitted for clarity. See Supporting Information Appendix S1 for model parameters

All three tested co‐variables exhibited significant influences on the allometric scaling of the ITD (Figure 2, Table 1). For bees, both GLMM and PGLMM analyses indicated that models including family or phylogeny and sex in interaction or in addition with the ITD, along with our nested random term better predicted dry weight relative to the baseline model (ITD‐only fixed effect model, model 10 (Table 1)) on the basis of K‐fold CV and Bayesian *R^2^* (Table 1; Δ*R^2^*: 0.046, Δ*K*‐fold CV: 2226.6). However, differences in K‐fold CV and Bayesian *R^2^*between the best‐fitting taxonomic and sexual dimorphism models were minimal (*R^2^* < 0.001; Δ*K*‐fold CV: 7.92), yet taxonomic models outperformed phylogenetic models in terms of *K*‐fold CV (Δ: 118.8) but not *R^2 ^*(Δ: 0.002). In hoverflies, incorporating sex and taxonomy increased body size predictions relative to the baseline ITD‐only models considerably (Δ*R^2^*: 0.058, Δ*K*‐fold CV: 80).

**Figure 3:**
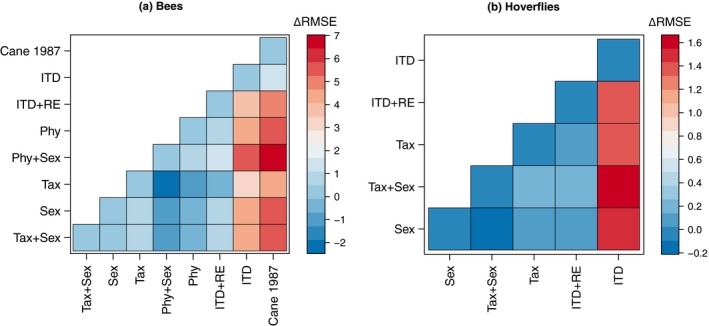
Pairwise comparisons of Δ root‐mean‐square error (RMSE) in milligrams between bee and hoverfly models. Blue values denote marginal precision differences in models, whereas red values indicate more error in models in the rows relative to the columns. Tax + Sex: Full taxonomic model, Tax: Reduced taxonomic model, Sex: Sexual dimorphic model, Phy + Sex: Full phylogenetic model, Phy: Reduced phylogenetic model, ITD + RE: ITD mixed effect model, ITD: ITD fixed effect model. Cane 1987: Cane (1987)'s)’s original model for bees

Reductions in predictive error as a result of incorporating co‐variates were most pronounced in bees in terms of root‐mean‐square error (RMSE) (Figure 3). All formulated models outperformed ITD‐only models in their predictive precision. The RMSE ranged between 10.228–12.427 (mg) for both taxonomic and phylogenetic GLMMs. The RMSE for the baseline ITD‐only model was 15.565 mg, which was near‐identical to the RMSE for Cane's (1987) original model: 15.553 mg. The RMSE for GLMMs for hoverflies ranged from 4.648 mg to 4.885 mg and all were slightly lower than the RMSE of the baseline ITD‐only model (6.169 mg). The range of prediction error for the ITD was also considerably lower than any pre‐existing and applicable model using body length: 36.36 mg ± 8.29 for bees and 7.99 mg ± 0.69 for hoverflies.

### 3.2 Intraspecific predictions

**Figure 4:**
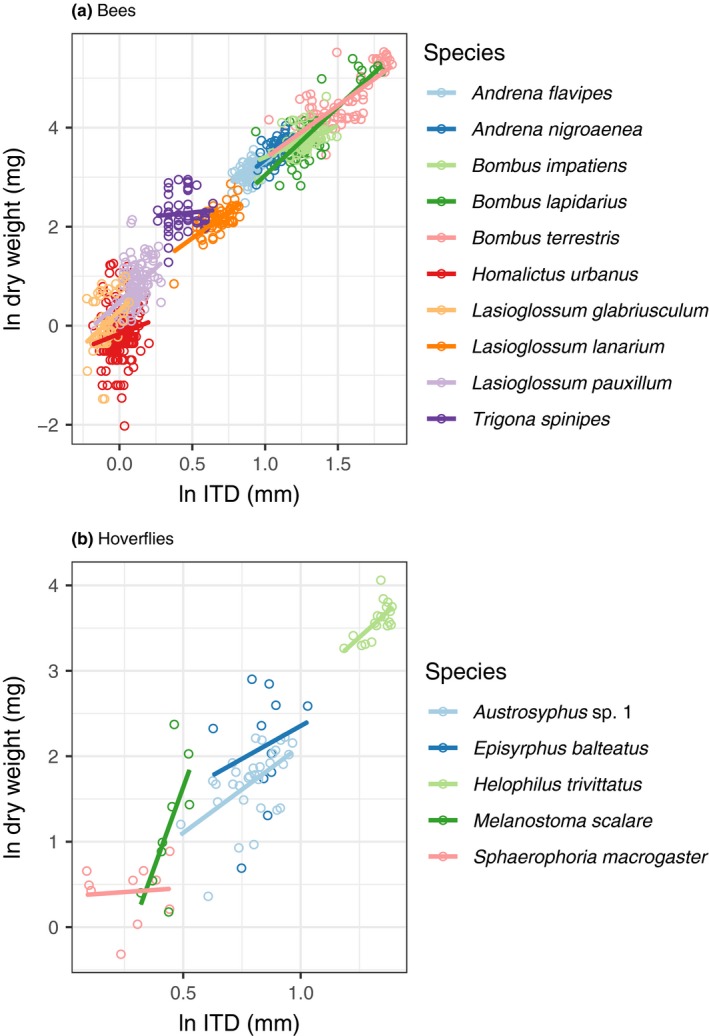
Intraspecific predictions of female* dry weight as a function of the intertegular distance (ITD). Lines denote line of best fit from OLS regression. *Except for *Bombus impatiens*

Across the 10 most abundant species of bees (♀ *Andrena flavipes*, ♀ *A. nigroaenea, *♂ *Bombus impatiens, *♀ *B. lapidarius,* ♀ *B. terrestris, *♀ *Homalictus urbanus, *♀ *Lasioglossum glabriusculum, *♀ *L. lanarium,* ♀ *L. pauxillum *and ♀ *Trigona spinipes*) and five most abundant hoverflies (♀ *Austrosyrphus *sp. 1*, *♀ *Episyrphus balteatus, *♀ *Helophilus trivittatus, *♀ *Melanostoma scalare, *and ♀ *Sphaerophoria macrogaster*), the strength of intraspecific predictions of body size using the ITD varied considerably (Table 2; Figure 4). All bee species exhibited a significant dry weight ~ ITD relationship, however, the adjusted‐*R^2^* differed considerably from 0.02 in *Homalictus urbanus* to 0.66 for *B. lapidarius*. Similarly, three of five hoverfly species, *Austrosyrphus* sp. 1, *H. parallelus, and M. scalare* exhibited a significant dry weight ~ ITD relationship. In order to accurately determine mean ITD and dry weight values for bees, a sample size of 20–30 specimens is required for trait values to stabilize within the 95% confidence intervals of the total sample size (see Supporting Information Figure S2).

**Table 2:** Model parameters of intraspecific ln dry weight—ln intertegular distance (ITD) relationships

Taxa	Region	Taxonomic ranking	Species	*F*_(_ *_df_*_)_	*α*	*β*	*R^2^*	P
Bee	Europe	Andrenidae: Andreninae	*♀ Andrena flavipes*	17.63 _(1,70)_	1.575 ± 0.367	1.73 ± 0.412	0.189	<0.001
	Europe	Andrenidae: Andreninae	*♀ Andrena nigroaenea*	30.17 _(1,50)_	0.893 ± 0.488	2.459 ± 0.448	0.364	<0.001
	North America	Apidae: Apinae	*♂ Bombus impatiens*	20.14 _(1,66)_	2.128 ± 0.365	1.275 ± 0.284	0.222	<0.001
	Europe	Apidae: Apinae	*♀ Bombus lapidarius*	110.2 _(1,54)_	0.277 ± 0.343	2.761 ± 0.263	0.665	<0.001
	Europe	Apidae: Apinae	*♀ Bombus terrestris*	137.8 _(1,81)_	1.242 ± 0.274	2.136 ± 0.182	0.625	<0.001
	Australia	Halictidae: Halictinae	*♀ Homalictus urbanus*	6.055 _(1,209)_	−0.164 ± 0.033	1.166 ± 0.474	0.024	0.014
	Europe	Halictidae: Halictinae	*♀ Lasioglossum glabriusculum*	6.444 _(1,47)_	0.302 ± 0.127	2.802 ± 1.104	0.102	0.014
	Europe	Halictidae: Halictinae	*♀ Lasioglossum lanarium*	53.87 _(1,61)_	0.702 ± 0.198	2.13 ± 0.29	0.46	<0.001
	Europe	Halictidae: Halictinae	*♀ Lasioglossum pauxillum*	37.46 _(1,129)_	0.488 ± 0.057	2.715 ± 0.444	0.219	<0.001
	South America	Apidae: Apinae	*♀ Trigona spinipes*	0.285 _(1,48)_	2.144 ± 0.243	0.287 ± 0.537	−0.02	0.596
Hoverfly	Australia	Syrphidae: Syrphinae	*♀ Austrosyrphus *sp. 1	12.7 _(1,30)_	0.087 ± 0.458	2.032 ± 0.57	0.274	0.001
	Europe	Syrphidae: Syrphinae	*♀ Episyrphus balteatus*	0.08 _(1,8)_	1.334 ± 1.885	0.885 ± 2.229	−0.11	>0.1.
	Europe	Syrphidae: Eristalinae	*♀ Helophilus trivittatus*	14.84 _(1,17)_	0.286 ± 0.857	2.485 ± 0.645	0.435	0.001
	Europe	Syrphidae: Syrphinae	*♀ Melanostoma scalare*	6.38 _(1,7)_	−2.172 ± 1.324	7.619 ± 3.016	0.4	0.03
	Australia	Syrphidae: Syrphinae	*♀ Sphaerophoria macrogaster*	0.04 _(1,8)_	0.361 ± 0.274	0.195 ± 0.907	−0.11	>0.1.

Note

*F*: *F*‐statistic and degrees of freedom for each model; *α*: intercept; *β*: ITD coefficients ± *SE*; *R^2^*: Adjusted *R^2^*; *p*: *p*‐value.

## 4 DISCUSSION

We present the most comprehensive examination of allometric scaling using intertegular distances (ITD), intraspecific variation, phylogenetic relatedness and biogeography to predict body size for two focal pollinating insect taxa: bees and hoverflies. We propose an iterative framework to develop and test this suite of highly predictive models for estimating body size in relation to phylogenetic relatedness and biogeographic differentiation. We also identified body size variation in the ITD due to sexual dimorphism (Bosch & Vicens, 2002; Skandalis, Tattersall, Prager, & Richards, 2009).

Sex was retained as an integral predictor either in addition or in interaction with the ITD for both taxa. This is likely because sexual size dimorphism (SSD) is common among insects. In both Diptera and Hymenoptera, up to 80% of species exhibit female‐biased SSD (i.e., larger females than males), (Shreeves & Field, 2008). Female‐biased SSD is hypothesized to be a result of increased fecundity as a result of larger female body size (Stillwell, Blanckenhorn, Teder, Davidowitz, & Fox, 2010). In bees, female‐biased SSD is attributed to the physical requirements of nest provisioning and construction (Shreeves & Field, 2008). In hoverflies, SSD was also notably female‐biased and sex was retained as the most important body size predictor in conjunction with the ITD. In both taxa, including sex increased model precision, but did not drastically change the overall body size prediction, highlighting the predictive accuracy of the ITD even when sex is not considered. Therefore, failing to incorporate sex in predictions will only introduce a subtle error. However, sex is easily identifiable in both bees and hoverflies (e.g., Michener, 2007 for bees and Stubbs & Falk 1983 for hoverflies). Therefore, we recommend its inclusion if body size predictions are used, as many ecologically relevant allometric traits are sex‐related (e.g., flight distances; Kraus, Wolf, & Moritz, 2009).

Few previous studies have assessed the utility of predictive models in describing intrageneric or intraspecific allometric traits (e.g., Hagen & Dupont, 2013, Cariveau et al., 2016a). Intraspecific). Intraspecific body size variation is difficult to predict accurately using co‐varying traits such as the ITD. In particular, the large variation in predictive power suggests that it is sensitive to environmental conditions and/or sample sizes. Adult body size variation, including co‐varying morphological traits (i.e., the ITD), in holometabolous insects is a direct result of diet and environment during ontogeny and larval development (Davidowitz, D'Amico, & Nijhout, 2004). For example, intraspecific body size variation has been attributed to seasonal variability and colony population increases in *Xylocopa virginica *and *Bombus *spp. (Inoue, 1992; Skandalis et al., 2009). Therefore, dietary differences, gut contents and starvation periods, for which we did not account for, likely cause intraspecific variability in the body size ~ ITD relationship.

These intraspecific patterns raise the question of how many individuals are necessary to measure to accurately capture species’ mean trait values. Our analyses suggest that 20–30 specimens per species will provide accurate estimates of intraspecific body size, the ITD and potentially other morphological trait values.

Incorporating phylogenetic information is a cornerstone of comparative biological analyses. Phylogenetic signal in body size variation has been inferred in a number of vertebrate and invertebrate groups (Ashton, 2004). Failing to account for dependent phylogenetic patterns can lead to inaccurate predictions (Garland, Bennett, & Rezende, 2005; Martins & Housworth, 2002). In our study, both PGLMM and GLMM models were comparable in terms of predictive power. Interestingly, taxonomic and phylogenetic GLMM models were near‐identical in both Bayesian *R^2^* and RMSE demonstrating that differential allometric scaling is present at/or below the family level. These results suggest that predictive inferences of body size that don't account for evolutionary history lack accuracy and generalizability.

Where the aim is prediction, GLMMs incorporating taxonomic groupings without considering phylogeny are more practical. First, well‐resolved phylogenies are lacking for most groups and second, taxonomy‐based models allow us to predict allometric relationships for nonrepresented species, while phylogenetic models are only applicable to species contained within the used phylogeny. A further advantage of using taxonomic groupings over phylogeny is that they provide easy‐to‐interpret regression intercepts and/or slopes as opposed to a phylogenetic covariance matrix. For bees, we confirm that incorporating taxonomy is predictively equivalent to including phylogenetic information in allometric scaling relationships where the latter is unavailable. This uniformity between taxonomic and phylogenetic models may not exist for other taxa with either high paraphyly, low correspondence between taxonomy and phylogeny or for other nontested allometric biological traits. In hoverflies, including taxonomy was less informative than for bees, potentially due to the lower taxonomic ranking used (i.e., subfamily).

By simulating infrageneric polytomies within our phylogeny, we implemented a conservative approach which does not fully recognize the true infrageneric phylogenetic structure. Although infrageneric phylogenies exist for some genera (e.g., *Bombus* and *Lasioglossum*, Cameron, Hines, & Williams, 2007; Danforth, Conway, ‐ Ji, 2003), these were not available for the majority of incorporated bee species. However, we posit that the effect of these would be minimal, relative to the total interspecific branch lengths between congeneric species. However, it may exhibit a stronger influence on more closely related species, or within those genera that have multiple subgenera (e.g., *Lasioglossum*, Michener, 2007). Future studies should attempt to incorporate known infrageneric branch lengths in order to more accurately account for these patterns.

Terrestrial invertebrates show considerable biogeographic variation in body shape and size. While previous studies have compared allometric models between biogeographical regions either independently (Schoener, 1980) or within a meta‐analytical framework (Martin et al., 2014), we chose to represent biogeographical variation within a random effect structure. This makes these models broadly applicable and not biogeographically restricted in utility. Observed biogeographical differences within this study likely arise from differing species diversification patterns as well as from sampling biases, such as variation in commonality among species. Therefore, it is problematic to disentangle and prove hypotheses that explain biogeographic variation in the allometric scaling of the ITD. However, it is clear that the influence of biogeography appears alongside species’ evolutionary histories and intraspecific variation.

The structure of our study design had several limitations. First, there is a potential measurement error as a result of multiple contributors measuring and weighing specimens. Second, specimen condition may introduce subtle errors, for example, due to the presence of pollen loads in species which removal is often difficult or impossible (e.g., for bees which collect it internally, e.g., *Hylaeus* spp., Scott, 1996). Finally, sampling variation attributable to low specimen sample sizes or specimens for which we only have a single gender may have introduced some error. Greater accuracy will likely be achieved in the iterative process of updating these models as new data become available.

By incorporating sexual dimorphism, phylogeny or taxonomy, and biogeography we improved model predictions and reduced the limitations of traditional allometric models used to estimate body size. These three predictors represent fundamentally related causes of body size variation in pollinating insects. In consideration of the multiple metrics (i.e., Bayesian *R^2^*, K‐fold CV, and RMSE) used in model selection,, we provide multiple predictive models. This is important as research questions may not focus on sex‐related allometric differences and may occur outside the included biogeographic regions or taxonomic groups (i.e., the bee family Stenotritidae or the hoverfly subfamily Microdontinae). Therefore, disseminating the most appropriate allometric model becomes a hypothesis‐driven formula that should consider and then discount each examined factor. Importantly, given the high resolution of our described models and the large sample size of specimens within this study, our models improve body size predictions relative to pre‐existing models even when considering only the ITD. After accounting for biogeographical and species‐level effects, failing to incorporate sex or phylogeny/taxonomy will not result in considerable error (see Figure 3) although we endorse their use as it enables more meaningful analyses. Lastly, we caution the use of ordinal‐level predictive models as allometric constraints differ considerably at the family level (see Figure 1).

### 4.1 Summary of R package functions

The developed *R* package, “*pollimetry,”* integrates models for estimating body size (i.e., dry weight) in bees and hoverflies using the ITD and co‐variates (Table 1). These models were collated, using the enclosed dataset, into a single function that returns body size estimates, standard error, and 90% credible intervals, based on the user's model choice. In addition, *pollimetry* includes functions for estimating pollinator dry weight using pre‐existing models that utilize the following co‐varying traits: body length, head width, and body length * body width; see Supporting Information Appendix S1). The *R* package also includes functions for estimating bee foraging distances using the ITD (Greenleaf et al., 2007) or head width (van Nieuwstadt & Iraheta, 1996). We reimplemented (Cariveau et al., 2016a, 2016b) predictive models for bee tongue length using the ITD and taxonomic family from the available raw data. We also included allometric functions to calculate bee field nectar load (Henry & Rodet, 2018) and wing loading (Bullock, 1999). These equations will be updated in future package releases as models are re‐fit to include new data.

### 4.2 Conclusions and implications

The accompanying *R* package, “*pollimetry,*” provides a user‐friendly interface to estimate pollinator body size (as dry weight) and co‐varying ecological traits. Practical allometric libraries require multiple models that will be updated when new datasets become available. This will enable robust investigation of other allometric traits at both intra‐ or interspecific levels. The consequences of body size variation are ubiquitous within pollination research, yet few have utilized allometric theory in studying pollinating taxa beyond bees. Providing more robust estimates of body size for bees and hoverflies is an important first step, yet this comprehensive approach to allometric model development should be applied to other pollinating taxa, such as Lepidoptera. The iterative framework developed herein, heralds a dynamic new direction for allometric models of body size and co‐varying ecological traits and will provide more accurate predictions through hypothesis‐led model choice, testing, and investigation in allometric research.

## AUTHOR CONTRIBUTIONS

IB, LKK, VG and RR conceived the study. LKK, VG, JR, and MH collected Australian specimens. BMF and JSP collected, identified, and measured the Brazilian bees. LKK measured Australian, German and Swiss specimens. LKK and MH identified Australian bees. ZMP identified North American specimens. AH collated German specimens. LR collected, identified, and measured Irish specimens. JMM collected and identified British specimens. FPM collected, identified, and measured Spanish specimens. NJV and SPMR collected, identified, and measured Belgian specimens. MA and LS collected Swiss specimens. LKK, IB, and VG devised and undertook all data analyses. LKK and IB formulated and wrote the R package. LKK wrote the manuscript and all authors contributed significantly to the final manuscript.

## DATA AVAILABILITY

All data including R code and the *R* package are available here: https://github.com/liamkendall/pollimetry ≤https://doi.org/10.5281/zenodo.1313905≥.
