# Supplementary material for: Pollinator size and its consequences: Robust estimates of body size in pollinating insects
Source: Ecol Evol. 2019 Feb 7;9(4):1702–14. doi: 10.1002/ece3.4835 (PMC6392396; doi:10.1002/ece3.4835)
Supplement: Supplementary file 1 [file ECE3-9-1702-s001.docx]

**Supporting Information**

*Description of* *pre-existing models*

In addition to developing new allometric models for bees and hoverflies, we selected the three key pollinating insect orders: Diptera, Hymenoptera and Lepidoptera and collated all known allometric models for those orders, regardless of whether they are acknowledged pollinators. Lepidoptera were not included in primary within-text analyses for logistic reasons and low abundances across sourced research projects. From an initial literature search, we obtained the publications analysed by Martin et al. (2014). We then reviewed each publication individually, including their references and citations for additional models.

Diptera: 26 allometric models for Diptera were collated (Table S1A). Eleven models were reported for the entire order, including nine without any taxonomic breakdown of samples used. Twelve models were collated for the three main suborders Nematocera (6), Brachycera (4) and Cyclorrhapha (2) and two for specific families; Asilidae and Bombyliidae.

Hymenoptera: 38 allometric models for Hymenoptera were collated (Table S1B). These included eight models for the entire order, ten for Formicidae and seven for all Hymenoptera excluding Formicidae. There are three models for Vespidae and two models for Apidae (Cane, 1987 & Sabo et al., 2002). Sample et al., (1993)’s body length and body length * body width models are provided for Braconidae, Ichneumonidae, Halictidae and Pompilidae. We also collated Cariveau et al., (2016)’s allometric equations for bee tongue length, Greenleaf et al., (2007)’s allometric equations for bee foraging distances, Bullock (1999)’s allometric equation for wing loading and Henry & Rodet (2018)’s

Lepidoptera: 21 allometric models for Lepidoptera were collated (Table S1C). This includes 13 with varying taxa and without lower classifications. Hodar (1997) provides specific models for Heterocera (moths) and Ropalocera (butterflies). Sample et al. (1993) provide body length and body length * body width models for Microlepidoptera and two moth families: Geometridae and Arctiidae.

**Table S1A.** Allometric models for Diptera. Measure denotes trait measurement (BL = Body length, BW = Body width). Reg = regression type(L = Linear regression. MA = Major axis regression or OLS = Ordinary Least Squares regression). Type denotes slope (EXP = exponential model, PF = power function). Models are present in the form of $y=\ln\left( \alpha\right)+\ln\left( \beta\right)*x$ unless Type noted with *. ** = Included body width as well as length.

| **Source** | **Tax. grouping** | **Sample size** | **Biog. region** | **Measure** | **Range (mm)** | **Reg.** | **Type** | **Model** | | | |
| --- | --- | --- | --- | --- | --- | --- | --- | --- | --- | --- | --- |
|  |  | **(Families: species)** |  |  |  |  |  | ***α* ± S.E.** | ***Β* ± S.E.** | **Resi. SE** | ***R^2^*** |
| Rogers et al., (1977) | NA | (NA:84) | USA | BL | 0.9-34 | OLS | PF | -3.298 ± 0.115 | 2.366 ± 0.078 | 0.57 | **0.96** |
| Schoener (1980) | NA | (NA:107) | Costa Rica | BL | NA | OLS | PF | -2.603 ± 0.0688 | 1.64 ± 0.1224 | NA | 0.80 |
| “ | NA | (NA:124) | “ | “ | “ | “ | “ | -2.688 ± 0.051 | 1.59 ± 0.1173 | NA | 0.78 |
| “ | NA | (NA:171) | USA | “ | “ | “ | “ | -3.816 ± 0.561 | 2.42 ± 0.0969 | NA | 0.89 |
| Gowing and Recher (1984) | NA | (NA:100) | Australia | BL | 2-11 | OLS | PF | 3.653 ± 0.129 | 2.546 ± 0.071 | 0.37 | **0.93** |
| Sample et al., (1993) | Combined | (15:257) | USA | BL | 2.9-23.65 | OLS | PF | -3.184 ± 0.184 | 2.213 ± 0.085 | NA | 0.85 |
| “ | “ | “ | “ | BL*BW | “ | “ | “ | -2.197 ± 0.089 | 1.309 ± 0.03 | “ | **0.94** |
| “ | BIB, SCI, TIP | (3:46) | “ | BL | 3.55-23.65 | “ | “ | -3.675 ± 0.23 | 2.212 ± 0.141 | “ | **0.92** |
| “ | “ | “ | “ | BL*BW | “ | “ | “ | -2.217 ± 0.205 | 1.288 ± 0.071 | “ | **0.94** |
| “ | ASI, DOL, EMP, RHA, STR, THE | (6:80) | “ | BL | 2.9-17.99 | “ | “ | -3.374 ± 0.230 | 2.158 ± 0.101 | “ | **0.92** |
| “ | “ | “ | “ | BL*BW | “ | “ | “ | -2.2 ± 0.147 | 1.259 ± 0.049 | “ | **0.95** |
| “ | CAL, LAU, MUS, OTI, SYR, TAC | (6:119) | “ | BL | 2.9-15.65 | “ | “ | -3.619 ± 0.212 | 2.632 ± 0.101 | “ | **0.92** |
| “ | “ | “ | “ | BL*BW | “ | “ | “ | -2.02 ± 0.131 | 1.298 ± 0.042 | “ | **0.94** |
| Hodar (1997) | BRA | (NA:26) | Spain | HW | NA | OLS | PF | 0.655 ± 0.105 | 2.526 ± 0.139 | 0.47 | **0.93** |
| **“** | NEM | (NA:10) | “ | “ | NA | “ | “ | 3.942 ± 0.259 | 3.106 ± 0.278 | 0.55 | **0.94** |
| Ganihar (1997) | NA | (NA:20) | India | BL | NA | OLS | PF | -3.4294 ± 0.01994 | 2.5943 ± 0.0334 | 0.03 | **0.99** |
| Johnson and Strong (2000) | NA | (NA:75) | Jamaica | BL | 1-12.5 | OLS | PF | -2.462 ± 0.196 | 1.881 ± 0.146 | NA | 0.83 |
| “ | NEM | (NA:21) | “ | “ | 1-4.8 | “ | “ | -2.562 ± 0.244 | 1.373 ± 0.207 | “ | 0.84 |
| “ | NEM exc. | (NA:54) | “ | “ | 1.2-12.5 | “ | “ | -2.105 ± 0.178 | 1.805 ± 0.124 | “ | 0.89 |
| Sabo et al., (2002) | BRA | NA | USA | BL | NA | OLS | PF* | 0.006 ± 0.007 | 3.05 ± 0.36 | NA | 0.85 |
| “ | NEM | NA | “ | “ | “ | “ | “ | 0.1 ± 0.06 | 1.57 ± 0.2 | “ | **0.90** |
| “ | ASI | (1:9) | “ | “ | “ | “ | “ | 0.38 ± 2.625 | 1.5 ± 2.469 | “ | 0.74 |
| “ | BOM | (1:10) | “ | “ | “ | “ | “ | 0.007 ± 0.011 | 3.337 ± 0.676 | “ | **0.95** |
| Brady and Noske (2006) | NA | (NA:9) | Australia | BL | 2-28 | OLS | L* | -0.041 ± 0.004 | 0.010 ± 0.001 | 0.02 | 0.84 |
| Wardhaugh (2013) | NA | (NA:16) | Australia | BL | NA | MA | PF | -3.29 ± 0.45 | 2.65 ± 0.36 | NA | 0.72 |
| “ | “ | “ | “ | BL * BW | NA | “ | “ | -1.91 ± 0.19 | 1.22 ± 0.11 | “ | 0.87 |

BIB =Bibionidae, SCI = Sciaridae, TIP = Tipulidae, ASI= Asilidae, BOM: Bombyliidae, DOL = Dolichopodidae, EMP = Empididae, RHA = Rhagionidae, STR = Stratiomyidae, THE = Therevidae, CAL = Calliphoridae, LAU = Lauxaniidae, MUS = Muscidae, OTI = Otitidae, SYR = Syrphidae, TAC = Tachinidae. NEM = Nematocera, BRA= Brachycera, CYC = Cyclorrapha

**Table S1B.** Allometric models for Hymenoptera. Measure denotes trait measurement (BL = Body length, BW = Body width, ITD = Intertegular distance). Reg = regression type (L = Linear regression. MA = Major axis regression or OLS = Ordinary Least Squares regression). Type denotes slope (EXP = exponential model, PF = power function). Models are present in the form of $y=\ln\left( \alpha\right)+ *ln(x)$ unless Type noted with *. ** = Included body width as well as length.

| **Source** | **Tax. grouping** | **Sample size** | **Biog. region** | **Measure** | **Range (mm)** | **Reg.** | **Type** | **Model** | | | |
| --- | --- | --- | --- | --- | --- | --- | --- | --- | --- | --- | --- |
|  |  | **(Families: species)** |  |  |  |  |  | ***α* ± S.E.** | ***Β* ± S.E.** | **Resi. SE** | ***R^2^*** |
| Rogers et al., (1977) | ** | (NA:97) | USA | BL | 0.7-27 | OLS | PF | -3.871 ± 0.108 | 2.407 ± 0.06 | 0.55 | **0.97** |
| “ | FOR | (NA:34) | “ | “ | 1.2-13.5 | OLS | PF | -4.029 ± 0.171 | 2.572 ± 0.097 | 0.4 | **0.98** |
| Cane (1987) | Apidae | (6:20) | USA | ITD | 1,6 | NL | PF | 0.6453 ± NA | 2.4691 ± NA | NA | **0.96** |
| Schoener (1980) | ** | (NA:174) | Costa Rica | BL | NA | OLS | PF* | 0.043 ± 0.05 | 2.07 ± 0.091 | NA | 0.87 |
| “ | “ | (NA:122) | “ | “ | “ | “ | “ | 0.022 ± 0.056 | 2.29 ± 0.137 | “ | 0.84 |
| “ | “ | (NA:82) | USA | BL | “ | “ | “ | 0.016 ± 0.072 | 2.55 ± 0.107 | “ | 0.94 |
| “ | FOR | (NA:25) | Costa Rica | BL | “ | “ | “ | 0.012 ± 0.113 | 2.72 ± 0.26 | “ | **0.91** |
| “ | “ | (NA:20) | “ | BL | “ | “ | “ | 0.21 ± 0.127 | 2.31 ± 0.224 | “ | **0.93** |
| “ | “ | (NA:13) | USA | BL | “ | “ | “ | 0.034 ± 0.155 | 2.19 ± 0.342 | “ | **0.91** |
| Gowing and Recher (1984) | ** | (NA:86) | Australia | BL | 1-12 | OLS | EXP | -2.860 ± 0.099 | 0.478 ± 0.016 | 0.48 | **0.92** |
| “ | FOR | (NA:68) | “ | “ | 2-18 | OLS | PF | -3.306 ± 0.258 | 2.489 ± 0.051 | 0.32 | **0.97** |
| Sample et al., (1993) | ALL | (7:274) | USA | BL | 2.81-34.91 | OLS | PF | -4.284 ± 0.183 | 2.696 ± 0.083 | NA | 0.89 |
| “ | “ | “ | “ | BL * BW | “ | “ | “ | -2.375 ± 0.08 | 1.456 ± 0.028 | NA | **0.95** |
| “ | Ichneumonidae | (1: 106) | “ | BL | 3.65-34.91 | “ | “ | -4.149 ± 0.262 | 2.464 ± 0.116 | NA | **0.9** |
| “ | “ | “ | “ | BL * BW | “ | “ | “ | -2.497 ± 0.147 | 1.445 ± 0.053 | NA | **0.94** |
| “ | Braconidae | (1:41) | “ | BL | 2.81-15.42 | “ | “ | -3.854 ± 0.273 | 2.441 ± 0.147 | NA | **0.94** |
| “ | “ | “ | “ | BL * BW | “ | “ | “ | -2.19 ± 0.142 | 1.445 ± 0.069 | NA | **0.96** |
| “ | Vespidae | (1:19) | “ | BL | 8.14-20.58 | “ | “ | -3.540 ± 0.544 | 2.782 ± 0.195 | NA | **0.96** |
| “ | “ | “ | “ | BL * BW | “ | “ | “ | -1.537 ± 0.307 | 1.319 ± 0.07 | NA | **0.98** |
| “ | Formicidae | (1:45) | “ | BL | 3.62-17.41 | “ | “ | -4.727 ± 0.350 | 2.919 ± 0.11 | NA | **0.93** |
| “ | “ | “ | “ | BL * BW | “ | “ | “ | -2.378 ± 0.265 | 1.473 ± 0.106 | NA | **0.9** |
| “ | Halictidae | (1:21) | “ | BL | 6-12.76 | “ | “ | -2.891 ± 0.386 | 2.302 ± 0.182 | NA | **0.95** |
| “ | “ | “ | “ | BL * BW | “ | “ | “ | -2.758 ± 0.357 | 1.590 ± 0.119 | NA | **0.95** |
| “ | Pompilidae | (1:15) | “ | BL | 5.55-14.32 | “ | “ | -2.341 ± 0.873 | 2.006 ± 0.396 | NA | 0.81 |
| “ | “ | “ | “ | BL * BW | “ | “ | “ | -1.946 ± 0.431 | 1.444 ± 0.154 | NA | **0.93** |
| Hodar (1997) | ALL | NA | Spain | HW | NA | OLS | PF* | 1.999 ± 0.112 | 2.09 ± 0.132 | 0.51 | **0.92** |
| “ | FOR – Workers | NA | “ | “ | “ | “ | “ | 0.552 ± 0.068 | 2.550 ± 0.116 | 0.19 | **0.98** |
| “ | FOR –Winged | NA | “ | “ | “ | “ | “ | 1.607 ± 0.127 | 2.752 ± 0.25 | 0.31 | **0.94** |
| Ganihar (1997) | NA** | (NA:26) | India | BL | NA | OLS | PF | -3.5917 ± 0.1646 | 2.6429 ± 0.1127 | 0.24 | **0.94** |
| Johnson and Strong (2000) | ALL | NA | Jamaica | BL | 1.4-24.3 | OLS | PF | -3.556 ± 0.183 | 2.193 ± 0.110 | NA | **0.92** |
|  | FOR | NA | “ | “ | 1.6-9.9 | “ | “ | -3.730 ± 0.298 | 2.103 ± 0.238 | “ | **0.9** |
|  |  | NA | “ | “ | 1.4-24.3 | “ | “ | -3.295 ± 0.241 | 2.102 ± 0.132 | “ | **0.92** |
| Sabo et al., (2002) | 7**** | (7:54) | USA | BL | NA | OLS | PF* | 0.56 ± 0.64 | 1.56 ± 0.4 | NA | 0.75 |
| “ | API | (1:10) | “ | “ | “ | “ | “ | 0.006 ± 0.041 | 3.407 ± 2.471 | “ | 0.81 |
| “ | VES | (1:19) | “ | “ | “ | “ | “ | 0.001 ± 0.002 | 3.723 ± 0.798 | “ | 0.95 |
| Brady and Noske (2006) | FOR | (NA:8) | Australia | BL | 2-10 | OLS | PF* | 0.001 | 2.330 ± 0.0151 | 0.49 | 0.71 |
| “ | ** | (NA:9) | “ | BL | 4-29 | OLS | PF* | 6.783 ± 0.001 | 2.544 ± 0.26 | 0.57 | 0.79 |
| Wardhaugh (2013) | NA | (NA:26) | Australia | BL | NA | MA | PF | -4.3 ± 0.38 | 3 ± 0.24 | NA | 0.83 |
| “ | “ | “ | “ | BL * BW | “ | “ | “ | -2.1 ± 0.09 | 1.34 ± 0.05 | “ | **0.97** |

API = Apidae, CHR = Chrysididae, FOR = Formicidae, ICH = Ichneumonidae, SPH = Sphecidae, VES = Vespidae. ** = excluded ants. ****Six families = API, CHR, FOR, ICH, SPH, VES

**Table S1C.** Allometric models for Lepidoptera. Measure denotes trait measurement (BL = Body length, BW = Body width). Reg = regression type (MA = Major axis regression, OLS = Ordinary Least Squares regression). Type denotes slope (EXP = exponential model, PF = power function). Models are present in the form of $y=\ln\left( \alpha\right)+\ln\left( \beta\right)*x$ unless Type noted with *. ** = Included body width as well as length.

| **Source** | **Tax. grouping** | **Sample size** | **Biog. region** | **Measure** | **Range (mm)** | **Reg.** | **Type** | **Model** | | | |
| --- | --- | --- | --- | --- | --- | --- | --- | --- | --- | --- | --- |
|  |  | **(Families: species)** |  |  |  |  |  | ***α* ± S.E.** | ***Β* ± S.E.** | **Resi. SE** | ***R^2^*** |
| Rogers et al., (1977) | NA | (NA:22) | USA | BL | 1.6-17 | OLS | PF | -4.037 ± 0.133 | 2.903 ± 0.08 | 0.31 | **0.99** |
| Sample et al., (1993) | ALL | (NA:384) | USA | BL | 2.76-40.73 | OLS | PF | -5.036 ± 0.157 | 3.122 ± 0.064 | NA | **0.93** |
| “ | “ | “ | “ | BL * BW | “ | “ | “ | -2.607 ± 0.088 | 1.457 ± 0.024 | “ | **0.95** |
| “ | MIC. | (NA:46) | “ | BL | 2.76-10.6 | “ | “ | -4.913 ± 0.325 | 2.918 ± 0.169 | “ | **0.93** |
| “ | “ | “ | “ | BL * BW |  | “ | “ | -2.715 ± 0.199 | 1.395 ± 0.08 | “ | **0.93** |
| “ | GEO | (1:58) | “ | BL | 6.45-21.70 | “ | “ | -4.172 ± 0.411 | 2.628 ± 0.167 | “ | **0.9** |
| “ | “ | “ | “ | BL * BW | “ | “ | “ | -2.343 ± 0.283 | 1.387 ± 0.084 | “ | **0.91** |
| “ | ARC | (1:60) | “ | BL | 5.05-20.06 | “ | “ | -3.755 ± 0.242 | 2.658 ± 0.105 | “ | **0.96** |
| “ | “ | “ | “ | BL * BW | “ | “ | “ | -1.658 ± 0.148 | 1.222 ± 0.044 | “ | **0.96** |
| Hodar (1996) | HET | (NA:10) | Spain | HW | NA | OLS | PF | 2.053 ± 0.25 | 2.804 ± 0.236 | 0.49 | 0.95 |
| “ | ROP | (NA:10) | “ | HW | NA | “ | “ | 1.634 ± 0.46 | 2.793 ± 0.446 | 0.48 | 0.83 |
| Ganihar (1997) | NA | (NA:10) | India | BL | NA | OLS | PF | -4.7915 ± 0.751 | 2.8585 ± 0.257 | 0.46 | **0.93** |
| Johnson and Strong (2000) | NA | (NA:40) | Jamaica | BL | 2.2-18.6 | OLS | PF | -3.268 ± 0.255 | 2.243 ± 0.130 | NA | **0.94** |
| Schoener (1980) | NA | (NA:29) | Costa Rica | BL | NA | OLS | PF* | 0.026 ± 0.186 | 2.55 ± 0.571 | NA | **0.96** |
| “ | “ | (NA:7) | “ | “ | “ | “ | “ | 0.078 ± 0.139 | 1.32 ± 0.683 | “ | 0.75 |
| “ | “ | (NA:18) | USA | “ | “ | “ | “ | 0.014 ± 0.18673 | 2.55 ± 0.571 | “ | 0.77 |
| Brady and Noske (2006) | NA | (NA:6) | Australia | BL | 7.34 | OLS | PF* | 0.001 | 2.313 ± 0.223 | 0.4 | 0.81 |
| Wardhaugh (2013) | NA | (NA:11) | Australia | BL | NA | MA | PF | -3.83 ± 0.41 | 2.77 ± 0.27 | NA | 0.83 |
| “ | “ | “ | “ | BL * BW | “ | “ | “ | -2.1 ± 0.21 | 1.37 ± 0.11 | “ | 0.88 |

HET = Heterocera, ROP = Ropalocera, MIC = Microlepidoptera, GEO = Geometridae, ARC = Arctiidae

**Table S2**. Allometric models for bee foraging distance, total field nectar load, tongue length and wing loading. BL: Body length (mm); HW: Head width (mm), ITD: Intertegular distance (mm).

| Type | Source | Taxa | Measurement | Model |
| --- | --- | --- | --- | --- |
| Foraging distance | Roubik and Aluja (1983) (in van Nieuwstadt & Iraheta (1996) | Apidae: Meliponini | Nectar source | $y=-579.1+550.9*HW$ |
|  | van Nieuwstadt & Iraheta (1996) |  | Nectar source | $y=-908.2+560.8*HW$ |
|  | Greenleaf et al., (2007) | Hymenoptera: Apoidea (excl. wasps) | Maximum | $y=-1.363 + 3.366*ln(ITD)$ |
|  |  |  | Typical | $y=-1.643 + 3.242*ln(ITD)$ |
|  |  |  | Feeder | $y=-0.760 + 2.313*ln(ITD)$ |
|  |  |  | Communication | $y=-0.993 + 2.788*ln(ITD)$ |
| Nectar load | Henry & Rodet (2018) | Hymenoptera: Apoidea (excl. wasps) | Total nectar load | $y=0.005*{BL}^{3.0618}$ |
| Bee tongue length | Cariveau et al., (2016) | Andrenidae | Glossa | $ln(y)=0.23 + 1.04*ln(ITD)$ |
|  |  | Apidae |  | $ln(y)=1.27 + 1.04*ln(ITD)$ |
|  |  | Colletidae |  | $ln(y)=0.21 + 1.04*ln(ITD)$ |
|  |  | Halictidae |  | $ln(y)=0.43 + 1.04*ln(ITD)$ |
|  |  | Megachilidae |  | $ln(y)=1.16 + 1.04*ln(ITD)$ |
|  |  | Andrenidae | Prementum | $ln(y)=0.88 + 0.83*ln(ITD)$ |
|  |  | Apidae |  | $ln(y)=0.91 + 0.73*ln(ITD)$ |
|  |  | Colletidae |  | $ln(y)=0.56 + 1.14*ln(ITD)$ |
|  |  | Halictidae |  | $ln(y)=0.89 + 1.04*ln(ITD)$ |
|  |  | Megachilidae |  | $ln(y)=0.77 + 0.68*ln(ITD)$ |
|  |  | Andrenidae | Proboscis | $ln(y)=1.06 + 0.96*ln(ITD)$ |
|  |  | Apidae |  | $ln(y)=2.13 + 0.96*ln(ITD)$ |
|  |  | Colletidae |  | $ln(y)=0.86 + 0.96*ln(ITD)$ |
|  |  | Halictidae |  | $ln(y)=1.38 + 0.96*ln(ITD)$ |
|  |  | Megachilidae |  | $ln(y)=1.87 + 0.96*ln(ITD)$ |
| Wing loading | Bullock (1999) | Hymenoptera: Apoidea (excl. wasps) | NA | $y=0.0119 + 0.668*ITD$ |

**References – Pre-existing models**

Brady, C. J. and Noske, R. A. (2006). Generalised regressions provide good estimates of insect and spider biomass in the monsoonal tropics of Australia.

*Australian Journal of Entomology* 45(3): 187-191.

Bullock, S. H. (1999). Relationships among body size, wing size and mass in bees from a tropical dry forest in Mexico. *Journal of the Kansas Entomological Society*, 426-439.

Cane, J. H. (1987). Estimation of bee size using intertegular span (Apoidea). *Journal of Kansas Entomological Society* 60(1): 145-147.

Cariveau, D. P., Nayak, G. K., Bartomeus, I., Zientek, J., Ascher, J. S., Gibbs, J., and Winfree, R. (2016). The allometry of bee proboscis length and its uses in ecology. PloS one, 11(3), e0151482.

Ganihar, S. R. (1997). Biomass estimates of terrestrial arthropods based on body length. – *Journal of Bioscience* 22(2): 219-224.

Gowing, G. and Recher, H. F. (1984). Length-weight relationships for invertebrates from forests in south-eastern New South Wales. *Austral Ecology* 9(1): 5-8.

Greenleaf, S.S., Williams, N.M., Winfree, R. and Kremen, C., (2007). Bee foraging ranges and their relationship to body size. *Oecologia* 153(3): 589-596.

Henry, M., and Rodet, G. (2018). Controlling the impact of the managed honeybee on wild bees in protected areas*. Scientific reports* 8(1), 9308.

Hodar, J. A. (1997). The use of regression equations for the estimation of prey length and biomass in diet studies of insectivore vertebrates. *Miscellania Zoologica* 20(2): 1-10.

Johnson, M. D. and Strong, A. M. (2000). Length-weight relationships of Jamaican arthropods. *Entomological News* 111(4): 270-281.

Rogers, L., Buschbom, R. and Watson, C. (1977). Length-weight relationships of shrub-steppe invertebrates. *Annals of the Entomological Society of America* 70(1): 51-53.

Sabo, J. L., Bastow, J. L. and Power, M. E. (2002). Length-mass relationships for adult aquatic and terrestrial invertebrates in a California watershed. *Journal of the North American Benthological Society* 21(2): 336-343.

Sample, B.E., Cooper, R.J., Greer, R.D. and Whitmore, R.C., (1993). Estimation of insect biomass by length and width. *The American Midland Naturalist* 129(2): 234-240.

Schoener, T. W. (1980). Length-weight regressions in tropical and temperate forest-understory insects. *Annals of the Entomological Society of America* 73(1): 106-109.

Stubbs, A. E. & Falk, S. J. (1983). *British hoverflies: An illustrated identification guide*. Reading, UK: British Entomological and Natural History Society.

van Nieuwstadt, M. and Iraheta, C. R. (1996). Relation between size and foraging range in stingless bees (Apidae, Meliponinae). – Apidologie. 27: 219-228. *Apidologie* 27(4): 219-228.

Wardhaugh, C. W. (2013). Estimation of biomass from body length and width for tropical rainforest canopy invertebrates. *Australian Journal of Entomology* 52(4): 291-298.

**Table S3**. Distribution of included specimens. Numbers in parenthesis denote total specimens and species per country, family and/or subfamily. Exact sampling locations are available in the included dataset. ID: Specimen identifier. Either study author initials or full name and affiliation. DL: Specimen deposition location. Letters in superscript refer to author affiliations or institution address is provided. * All excluding Jim Cane’s specimens (see Cane, 1987).

| Taxa | Region | Country | Family | Subfamily | ID | | DL |  |
| --- | --- | --- | --- | --- | --- | --- | --- | --- |
| Bee | | Australasia | Australia (899, 93) | Apidae (185, 20)  Colletidae (130, 15)  Halictidae (519, 38)  Megachilidae | Apinae (125, 13),Xylocopinae (60, 7)  Colletinae (76, 7), Euryglossinae (49, 3)  Hylaeinae (5, 5)  Halictinae (441, 25)  Nomiinae (78, 13)  Megachilinae (65, 20) | | LKK & MH | UNER; MHPC |
|  | Europe | Belgium (703, 49) | Andrenidae  Apidae (242)  Halictidae  Megachilidae  Melittidae (14) | Andreninae (253, 15)  Apinae (192, 9), Nomadinae (50, 4)  Halictinae (120, 9)  Megachilinae (74, 9)  Dasypodainae (3, 1), Melittinae (11, 2) | NJV, SPMR & Alain Pauly^A^ | | ULBC |  |
|  |  | Germany (765, 63) | Andrenidae  Apidae (189, 12)  Colletidae  Halictidae  Megachilidae | Andreninae (197, 14)  Apinae (188, 11), Nomadinae (1, 1)  Hylaeinae (13, 6)  Halictinae (337, 18)  Megachilinae (29, 13) | Klaus Mandery^B^ | | KMIB |  |
|  |  | Ireland (52, 15) | Andrenidae  Apidae  Colletidae  Halictidae | Andreninae (4, 2)  Apinae (29, 6)  Hylaeinae (5, 2)  Halictinae (14, 5) | LR | | TCDS |  |
|  |  | Spain (74, 46) | Andrenidae (18, 13)  Apidae (27, 17)  Colletidae  Halictidae (11, 6)  Megachilidae  Melittidae | Andreninae (10, 8), Panurginae (8, 5)  Apinae (16, 10), Nomadinae (6, 4)  Xylocopinae (5, 3)  Colletinae (4, 2)  Halictinae (8, 5), Rophitinae (3, 1)  Megachilinae (7, 6)  Dasypodainae (7, 2) | FPM & Oscar Aguado^H^ | | EBDS |  |
|  |  | Switzerland (210, 63) | Andrenidae  Apidae (60, 20)  Colletidae  Halictidae  Megachilidae  Melittidae | Andreninae (54, 14)  Apinae (54, 16), Nomadinae (4, 3), Xylocopinae (2, 1)  Colletinae (3, 1)  Halictinae (76, 20)  Megachilinae (15, 6)  Melittinae (2, 2) | Sonja Gerber^C^, Michael Herrmann^D^ and Andreas Müller^E^ | | AGZS |  |
|  |  | UK (46, 4) | Apidae | Apinae (46, 4) | JMM | | UNER |  |
|  | North America | USA (1082, 132) | Andrenidae (155, 35)  Apidae (378, 27)  Colletidae (86, 8)  Halictidae (396, 44)  Megachilidae (17)  Melittidae (1) | Andreninae (150, 30), Oxaeinae (1, 1), Panurginae (4, 4)  Apinae (195, 18), Nomadinae (17, 4), Xylocopinae (166, 4)  Colletinae (3, 2), Hylaeinae (83, 6)  Halictinae (241, 42), Nomiinae (1, 1), Rophitinae (1, 1)  Megachilinae (67, 17)  Dasypodainae (1, 1) | ZMP, Cane (1987) | | UMSP* |  |
|  | South America | Brazil (204, 22) | Andrenidae  Apidae (174, 17)  Halictidae  Megachilidae | Panurginae (8, 1)  Apinae (149, 12), Xylocopinae (25, 5)  Halictinae (11, 2)  Megachilinae (11, 2) | BMF, JSP | | UCFB |  |
| Hoverfly | | Australasia | Australia (120, 19) |  | Eristalinae (25, 7), Pipizinae (1,1), Syrphinae (94, 11) | | Susan Wright^F^ | UNER |
|  | | Europe | Ireland (39, 15) |  | Eristalinae (8, 5), Syrphinae (31, 10) | | LR | TCDS |
|  | Spain (8, 8) |  | Eristalinae (6, 6), Syrphinae (2, 2) | FPM & Oscar Aguado^H^ | | EBDS |  |  |
|  | | Switzerland (232, 79) |  | Eristalinae (114, 37), Pipizinae (12,5) Syrphinae (106, 37) | | Ruth Bärfuss^G^ | AGZS |  |

*Taxonomist affiliations*: **A**: Institut royal des Sciences naturelles de Belgique, O.D. Taxonomie & Phylogénie, Rue Vautier 29, 1000 Bruxelles, Belgium. **B**: Institut für Biodiversitätsinformation e.V. Geschwister-Scholl-Str. 6 96106 Ebern, Germany. **C**: Drosera Ecologie Appliquée SA Chemin de la Poudrière 36 1950 Sion Switzerland. **D**: WAB-Mauerbienenzucht Sonnentauweg 47 78467 Konstanz Germany. **E**: Natur Umwelt Wissen GmbH Bergstrasse 162 8032 Zürich Switzerland. **F**: Queensland Museum, PO Box 3300, South Brisbane BC, Queensland 4101, Australia. **G**. Ruth Bärfuss Feldstrasse 7 8625 Gossau ZH Switzerland. **H**: Freelance/no affiliation.

*Specimen deposition locations*: **AGZS**: Agroscope, Agroecology and Environment, Zürich, Switzerland. **EBDS**: Estación Biológica de Doñana Collection, Sevilla, Spain. **KMIB**: Klaus Mandery’s collection, Institut für Biodiversitätsinformation, Bern, Germany. **MHPC**: Mark Hall’s personal collection, Australia. **TCDS**: Stout Lab, Trinity College, Dublin, Ireland. **UCFB**: Bee Laboratory Collection, Federal University of Ceará, Fortaleza, Brazil. **ULBC**: Agroecology Lab reference collection, Université libre de Bruxelles (ULB), Belgium. **UMSP**: University of Minnesota Insect Collection, USA. **UNER**: Rader Lab Insect Collection, University of New England, Armidale, Australia.

**Taxonomic resources used within this study for identifying insect specimens**

Thompson, F.C., Rotheray, G.E., 1998. Family Syrphidae. In: Papp,

L., Darvas, B. (Eds.), Manual of Palaearctic Diptera, Vol. 3.

Science Herald, Budapest, pp. 8, 1–139.

Amiet, F., Herrmann, M., Müller, A. & Neumayer, R. (1996). Insecta Helvetica Fauna 12, Hymenoptera Apidae 1-9. Schweizerische Entomologische Gesellschaft.

Ascher, J. S. & Pickering, J. (2018). Discover Life bee species guide and world checklist (Hymenoptera: Apoidea: Anthophila). URL http://www.discoverlife.org/mp/20q?guide=Apoidea_species [Accessed 7 June 2018]

Atlas of Hymenoptera. (2017). URL <http://www.atlashymenoptera.net/liste_them.asp?them=Belgium> [accessed 20 January 2017]

Baker, J. R. (1975). Taxonomy of five nearctic subgenera of *Coelioxys* (Hymenoptera: Megachilidae). *University of Kansas Science Bulletin,* 50, 649–730.

Ball, S. & Morris, R. (2015). *Britain's Hoverflies: A Field Guide-Revised and Updated Second Edition*. Princeton, NJ: Princeton University Press.

Batley, M. & Houston, T.F. (2012). Revision of the Australian bee genus Trichocolletes Cockerell (Hymenoptera: Colletidae: Paracolletini). *Records of the Australian Museum*, 64, 1-50.

Bouseman, J. K. & LaBerge, W. E. (1978). A revision of the bees of the genus *Andrena* of the Western Hemisphere. Part IX. Subgenus Melandrena. *Transactions of the American Entomological Society*, 104, 275–389.

Coelho, B. W. T. (2004). A review of the bee genus *Augochlorella* (Hymenoptera: Halictidae: Augochlorini). *Systematic Entomology* 29, 282–323.

Dollin, A.E. and Dollin, L.J. (1997). Australian stingless bees of the genus *Trigona* (Hymenoptera: Apidae). *Invertebrate Systematics*, 11(6), 861-896.

Dollin, A.E., Dollin, L.J. & Rasmussen, C. (2015). Australian and New Guinean stingless bees of the genus *Austroplebeia* Moure (Hymenoptera: Apidae)—a revision. *Zootaxa*, 4047(1), 1-073.

Falk, S. J. (2015). *Field guide to the bees of Great Britain and Ireland*. Devon, UK: British Wildlife Publishing.

Gibbs, J. (2011). Revision of the metallic *Lasioglossum* (Dialictus) of eastern North America (Hymenoptera: Halictidae: Halictini). *Zootaxa* 3073, 1–216.

Gibbs, J., Packer, L., Dumesh, S. & Danforth, B. (2013). Revision and reclassification of *Lasioglossum* (Evylaeus), L. (Hemihalictus) and L. (Sphecodogastra) in eastern North America (Hymenoptera: Apoidea: Halictidae). *Zootaxa* 3672, 1–117.

Gonzalez, V.H., Engel, M.S. & Griswold, T.L. (2013). The lithurgine bees of Australia (Hymenoptera: Megachilidae), with a note on *Megachile* *rotundipennis*. *Journal of Melittology*, (11), 1-19.

Houston, T.F. 91975). A revision of the Australian hylaeine bees (Hymenoptera: Colletidae). I. Introductory material and the genera *Heterapoides* Sandhouse, *Gephyrohylaeus* Michener, *Huleoides* Smith, *Pharohylaeus* Michener, *Hemirhiza* Michener, *Amphylaeus* Michener and *Meroglossa* Smith. *Australian Journal of Zoology Supplementary Series*, 23(36), 1-135.

Houston, T.F. (1981). A revision of the Australian hylaeine bees (Hymenoptera: Colletidae). II. *Australian Journal of Zoology Supplementary Series*, 29(80), 1-128.

Laberge, W. E. (1967). A revision of the bees of the genus *Andrena* of the Western Hemiphere. Part I Callandrena. (Hymenoptera:Andrenidae). *Bulletin of the University of Nebraska State Museum* 7, 1–316.

LaBerge, W. E. (1973). A revision of the bees of the genus *Andrena* of the Western Hemisphere. Part VI. Subgenus Trachandrena. *Transactions of the American Entomological Society* 99, 235–371.

Laverty, T. M. & Harder, L. D. (1988). The bumblebees of eastern Canada. *The Canadian Entomologist* 120, 965–987.

Leijs, R., Batley, M. & Hogendoorn, K. (2017). The genus *Amegilla* (Hymenoptera, Apidae, Anthophorini) in Australia: A revision of the subgenera Notomegilla and Zonamegilla. *ZooKeys*, 653, 79.

Maibach, A. Goeldlin de Tiefenau, P. & Dirickx, H. G. (1992). *Liste Faunistique des Syrphidae de Suisse (Diptera)*. Miscellanea Faunistica Helvetiae 1.

Mauss. V. (1994). Bestimmungsschlüssel für die Hummeln der Bundesrepublik Deutschland. – 5. Aufl. pp. 1-50. Deutscher Jugendbund für Naturbeobachtung.

Maynard, G.V. (2014). Revision of *Goniocolletes* and seven Australian subgenera of *Leioproctus* (Hymenoptera: Apoidea: Colletidae), and description of new taxa. *Zootaxa*, 3715, 1-114.

McGinley, R. J. (1986). Studies of Halictinae (Apoidea: Halictidae), I: Revision of new world Lasioglossum Curtis. *Smithsonian Contributions to Zoology* 429, 1–294.

Michener, C.D. (1965). A classification of the bees of the Australian and South Pacific regions. *Bulletin of the American Museum of Natural History*; v. 130.

Michener, C. D. (2007). *The bees of the world*. 2^nd^ Ed. Baltimore, MD: JHU press.

Miller, S. R., Gaebel, R., Mitchell, R.J. & Arduser M (2002). Occurrence of two species of old world bees, *Anthidium manicatum* and *A. oblongatum* (Apoidea: Megachilidae), in northern Ohio and southern Michigan. *Great Lakes Entomologist* 35, 65–69.

Mitchell, T. B. (1960). Bees of the eastern United States. I. *Technical bulletin (North Carolina Agricultural Experiment Station)*, 141, 1-538.

Mitchell, T.B. (1962). Bees of the eastern United States. II. *Technical bulletin (North Carolina Agricultural Experiment Station)*, 152, 1-557.

PaDIL: Australian pollinator database 2018. URL <http://www.padil.gov.au> [accessed 10 November 2017]

Ortiz-Sánchez, F. J. & Gallego, C. O. (2004). *Fauna ibérica. Vol. 23. Hymenoptera: Apoidea I*. Consejo Superior de Investigaciones Científicas (CSIC), Spain.

Patiny, S. & Terzo, M. (2010). *Catalogue et clé des sous-genres et espèces du genre Andrena de Belgique et du nord de la France (Hymenoptera, Apoidea)*. Université de Mons. Belgium.

Prys-Jones, O. E. & Corbet, S. A. (2011). *Bumblebees*. (3^rd^ ed.). Exeter, UK: Pelagic Publishing.

Rehan, S. M. & Sheffield, C. S. (2011). Morphological and molecular delineation of a new species in the *Ceratina dupla* species-group (Hymenoptera: Apidae: Xylocopinae) of eastern North America. *Zootaxa* 50, 35–50.

Reyes, S.G. (1993). Revision of the bee genus *Braunsapis* in the Australian region (Hymenoptera: Xylocopinae: Allodapini). *The University of Kansas science bulletin*, 55, 97-121.

Roberts, R. B. (1972). Revision of the bee genus *Agapostemon* (Hymenoptera: Halictidae). *University of Kansas Science Bulletin* 49, 437–590.

Roberts, R. B. (1973). Bees of northwestern America: *Halictus* (Hymenoptera: Halictidae). *Agricultural Experimental Station: Oregon State Technical Bulletin* 126, 23.

Röder, G. (1990). *Biologie der Schwebfliegen Deutschlands (Diptera: Syrphidae).* Erna Bauer Verlag, D-7538 Keltern-Weiler.

Scheuchl, E. (2000). *Illustrierte Bestimmungstabellen der Wildbienen Deutschlands und Österreichs. Band I: Anthophoridae. 2., erweiterte Auflage*. Eigenverlag.

Scheuchl, E. (2000). *Illustrierte Bestimmungstabellen der Wildbienen Deutschlands und Österreichs. Band II: Megachilidae – Melittidae*. Eigenverlag.

Schmid-Egger, C. & Scheuchl, E. (1997). *Illustrierte Bestimmungstabellen der Wildbienen Deutschlands und Österreichs. Band III: Andrenidae*. Eigenverlag.

Sheffield, C.S., Ratti, C., Packer, L. & Griswold, T. (2011). Leafcutter and mason bees of the genus *Megachile* Latreille (Hymenoptera: Megachilidae) in Canada and Alaska. *Canadian Journal of Arthropod Identification* 18, 1–107.

Silveira, F.A., Melo, G. A. & Almeida, E. A. B. (2002). Abelhas Brasileiras: Sistemática e identificação. 1 edição. edição do autor, Belo Horizonte.

Snelling, R. R. (1970). Studies on North American bees of the genus *Hylaeus*. 5. The subgenera Hylaeus, S. Str. and Paraprosopis (Hymenoptera: Colletidae*). Contributions in Science-Los Angeles County Museum* 180, 1–59.

Stubbs, A. E. & Falk, S. J. (1983). *British hoverflies: An illustrated identification guide*. Reading, UK: British Entomological and Natural History Society.

Terzo, M., Iserbyt, S. & Rasmont, P. (2007). Révision des Xylocopinae (Hymenoptera:Apidae) de France et de Belgique. *Annales de la Société entomologique de France (N.S.),* 43:4, 445-491

Walker, K.L. (1986). Revision of the Australian species of the genus *Homalictus* Cockerell (Hymenoptera: Halictidae). *Memoirs of the Museum of Victoria*, 47(2), 105-200.

Walker, K.L. (1995). Revision of the Australian native bee subgenus *Lasioglassum* (Chilalictus) (Hymenoptera: Halictidae). *Memoirs of the Museum of Victoria, 55*(2), 215 – 423.

Warncke, K. (1992). Die westpaläarktischen Arten der Bienengattung Sphecodes LATR. (Hymenoptera, Apidae, Halictinae). *Bericht Naturforschende Gesellschaft Augsburg* 52, 9-64.

**Table S4A**. Bees: Model parameters of best-fitting taxonomic GLMM and phylogenetic GLMM. Posterior: Posterior mean estimate (95% credible intervals). ESS: Effective sample size.Taxo-GLMM model formula: ln Dry weight ~ ln ITD + Family + Family:ln(ITD) + Sex + Sex:ln(ITD) + (1|Region/Species). Phylo-GLMM formula: ln Dry weight ~ ln ITD + Sex + Sex:ln(ITD) + (1|Region/Species).

| *Taxo.* GLMM | | | | | *Phylo*, GLMM | |
| --- | --- | --- | --- | --- | --- | --- |
| Effect type |  | Parameters | Posterior | ESS | Post. | ESS |
| Fixed | Intercept |  | 1.05 (0.64 – 1.37) | 873 | 0.81 (0.32 – 1.25) | 660 |
|  | lnITD |  | 2.09 (2.00 – 2.19) | 893 | 2.03 (1.94 – 2.11) | 1888 |
|  | Sex | Male | -0.1 (-0.15 – -0.05) | 2552 | -0.13 (-0.17 – -0.08) | 3020 |
|  |  | Male:lnITD | -0.15 (-0.21 – -0.09) | 3069 | -0.14 (-0.2 – -0.08) | 3263 |
|  | Family | Andrenidae | -0.08 (-0.27 – 0.10) | 879 | NA | NA |
|  |  | Colletidae | -0.33 (-0.52 – -0.13) | 629 | NA | NA |
|  |  | Halictidae | -0.35 (-0.49 – -0.21) | 632 | NA | NA |
|  |  | Megachilidae | -0.17 (-0.40 – 0.05) | 1024 | NA | NA |
|  |  | Melittidae | -0.47 (-0.97 – 0.03) | 2548 | NA | NA |
|  |  | Andrenidae:lnITD | 0.10 (-0.09 – 0.28) | 1265 | NA | NA |
|  |  | Colletidae:lnITD | -0.14 (-0.44 –0.13) | 1330 | NA | NA |
|  |  | Halictidae:lnITD | 0.02 (-0.15 –0.18) | 1195 | NA | NA |
|  |  | Megachilidae:lnITD | 0.02 (-0.22 – 0.26) | 1135 | NA | NA |
|  |  | Melittidae:lnITD | -0.01 (-0.21 –0.09) | 2688 | NA | NA |
| Random |  | Region | 0.29 (0.11 – 0.65) | 1702 | 0.27 (0.1 – 0.64) | 1752 |
|  |  | Region:Species | 0.30 (0.27 – 0.33) | 871 | 0.59 (0.52 – 0.66) | 977 |
|  |  | σ | 0.33 (0.32 – 0.34) | 4000 | 0.33 (0.32 – 0.34) | 4000 |

**Table S4B**. Hoverflies: Posterior mean model parameters for best-fitting GLMM. Post.: Posterior mean estimate (95% confidence intervals). ESS: Effective sample size. Model formula: ln dry weight ~ ln ITD + Sex + Sex:lnITD + (1|Region/Species).

| *Taxo*. GLMM | | | | |
| --- | --- | --- | --- | --- |
| Effect type |  |  | Post. | ESS |
| Fixed | Intercept |  | -0.21 (-0.93 – 0.23) | 1840 |
|  | lnITD |  | 2.52 (2.31 – 2.71) | 2672 |
|  | Sex | Male | -0.12 (-0.35 – 0.12) | 2811 |
|  |  | Male:lnITD | -0.09 (-0.35 – 0.17) | 2642 |
| Random |  | Region | 0.31 (0.07 – 0.79) | 2305 |
|  |  | Region:Species | 0.21 (0.13 – 0.29) | 1371 |
|  |  | σ | 0.44 (0.41 – 0.48) | 3555 |

**Fig. S1.** Photographs of A) intertegular distance (ITD) and B) body length (BL) measurements. Specimen is an Australian ♂ *Megachile (Eutricharaea) serricauda*.

**Fig. S2.** Intraspecific variation in intertegular distance (ITD) and body size (dry weight) in relation to sample size in the 10 most abundant bee species. Red lines denote the total trait mean and green lines represent 95% confidence intervals.
